# α-Linolenic acid-driven nano-liposomes from purslane seed oil modulate p-JAK2/p-STAT3 to combat acute liver failure

**DOI:** 10.1042/BSR20250383

**Published:** 2026-05-08

**Authors:** Shimaa O. Ali, Doaa Hegazy, Ibrahim S. Kamel, Naglaa H. Shalan, Asmaa A. ElMonier

**Affiliations:** 1 Department of Biochemistry, Faculty of Pharmacy, Cairo University, Cairo, Egypt; 2 Department of Pharmaceutics and Industrial Pharmacy, Faculty of Pharmacy, Cairo University, Cairo, Egypt; 3 Medical Laboratory program, Faculty of Applied Health Sciences, East Port Said National University, Port Said, Egypt; 4 Department of Pathology, Faculty of Medicine, Banha University, Banha, Egypt

**Keywords:** α-linolenic acid, Acute liver failure, JAK2, PPARγ, PSE-PMLs, STAT3, thioacetamide

## Abstract

Acute liver failure (ALF) is a serious illness characterized by extensive liver cell damage and inflammation. The present study highlights the potential of purslane seed oil extract-Pluronic-modulated liposomes (PSE-PMLs) to protect against thioacetamide (TAA)-induced ALF in rats. Four groups of rats were used in the present study: normal control (NC), TAA-induced ALF, silymarin (SIL), and PSE-PML groups. SIL- and PSE-PML-pretreated groups were orally pre-administered SIL (200 mg/kg) and PSE-PMLs (500 mg/kg), respectively, for 7 days. After these pretreatments, all groups except the NC group were injected intraperitoneally with TAA (350 mg/kg) to induce ALF. Serum levels of liver biochemical markers and inflammatory cytokines, interleukin-4, and interferon-gamma were estimated. Additionally, the hepatic malondialdehyde, superoxide dismutase activity, peroxisome proliferator-activated receptor-gamma coactivator 1-alpha, phosphorylated Janus kinase 2, phosphorylated signal transducer and activator of transcription 3, peroxisome proliferator-activated receptor-gamma, tumor protein p53, active cysteine-aspartic acid protease 3, and B-cell lymphoma 2 were assessed. A histopathological examination of the liver was also performed. The study found that pretreatment with PSE-PMLs mitigated TAA-induced biochemical and histopathological alterations, alleviating oxidative stress, dampening the overproduction of pro-inflammatory cytokines, and amending liver cell apoptosis. These pioneering findings suggest that PSE-PMLs are a promising prophylactic agent against ALF, warranting further studies in the post-injury models. The phytochemical profiling, gas chromatography–mass spectrometry, of purslane seed oil revealed α-linolenic acid (∼98%) as the predominant component, with additional sterols, tocopherols, and phenolic acids that may underlie its strong antioxidant and hepatoprotective effects.

## Introduction

Being a vital organ involved in various metabolic functions, the liver is frequently exposed to exogenous and endogenous injurious factors such as viruses, alcohol, drugs, chemicals, fats, and bio-transformed metabolites [1,2]. These factors are the main causative agents of hepatic injury and inflammation [3]. Acute liver failure (ALF) is a critical medical condition involving sudden loss of hepatic function, manifesting as severe coagulopathy, hepatic encephalopathy, and multi-organ failure [4].

Thioacetamide (TAA) is a widely used hepatotoxic agent to generate an experimental model for simulating ALF via mechanisms such as oxidative stress, inflammation, and cell death [5,6]. Metabolic products of TAA, such as TAA-S-S-dioxide and TAA-S-S-sulfoxide, are free radical derivatives that are responsible for inducing centrilobular necrosis, leading to damage of liver tissues [7].

Due to the severity of ALF and its limited treatment options, new and more effective therapeutic interventions are urgently needed. Many natural compounds are screened for their hepatoprotective activities against different types of hepatic damage, including TAA-induced hepatic injury [8–10]. Purslane (*Portulaca oleracea L*.) is a widespread succulent annual plant well-known to have antioxidant and anti-inflammatory activities; therefore, it has significant potential for attenuating liver injury caused by TAA toxins [11–14]. Purslane seed oil has significant bioactive potential. It is high in essential omega-3 and omega-6 fatty acids and phytosterols [15], which contribute to its protective effects in alleviating oxidative stress and inflammation. Limited data existed on individual lipid classes and sterols [16]. Moreover, these phytoconstituents are slowly absorbed because of their high molecular weights [11,17]. Therefore, the present study was intended to enhance the absorption of these phytoconstituents and thereby boost their bioavailability by integrating purslane in nanoparticles.

Owing to their merits, nanodrug carriers (NDCs) have attracted great attention for the delivery of different active substances through several administration routes, e.g., oral, topical, and intranasal routes. NDCs can enhance effective drug targeting to the site of action, protect drugs from physiological enzymes, reduce drug side effects, and increase the release sustainability of loaded drugs [18]. Liposomes are popular NDCs formed from a lipid bilayer enclosing an aqueous core. Owing to their structure, liposomes can carry both hydrophobic and hydrophilic drugs in the lipid bilayer and the aqueous core, respectively. This structure also imparts biocompatibility and biodegradability to liposomes. Unfortunately, the reported physical and chemical instability of liposomes limits their application. Consequently, several approaches, such as the incorporation of cholesterol and beta-sitosterol within the lipid bilayer, have been used to increase liposomal stability [19]. Pluronics, also called triblock copolymers, have been used as surfactants and stabilizers [20]. In addition to enhancing stability, Pluronic-modulated liposomes (PMLs) have been reported to increase *in vivo* drug delivery and intestinal drug absorption [21].

One of the major pathways for cytokine signal transduction is the Janus kinase/signal transducer and activator of transcription (JAK-STAT) signaling pathway, which is broadly involved in inflammation. Previously, it has been shown that JAK and STAT activation occurs widely throughout the liver in inflammatory cells. Specifically, as observed in the liver, cytokines trigger the activation of STAT3 mediated by JAK2. Upon receiving the signal from JAK2, STAT3 undergoes phosphorylation to form a dimer and subsequently translocates to the nucleus to modulate the transcription of associated genes. STAT3 primarily plays a role in regulating cell proliferation and differentiation, inflammation, and apoptosis [22,23]. In addition, it has been reported that hepatocyte necrosis is strictly connected to the JAK2/STAT3 signaling pathway [24]. Yet, the exact mechanisms of JAK2/STAT3 pathway inhibitors in liver injury remain largely unknown.

The hepatoprotective properties of purslane and α-linolenic acid are acknowledged, and nano-carriers have been investigated for hepatic delivery; however, the potential of purslane seed oil in a nano-liposomal pluronic-modulated system to moderate phosphorylated Janus kinase 2 (p-JAK2)/phosphorylated signal transducer and activator of transcription 3 (p-STAT3) and peroxisome proliferator-activated receptor-gamma coactivator 1-alpha (PGC1α)/peroxisome proliferator-activated receptor-gamma (PPARγ) signaling in TAA-induced ALF has yet to be examined. The present study also employed gas chromatography–mass spectrometry (GC-MS) profiling to characterize the phytochemical constituents of the crude oil.

## Materials and methods

### Materials

Egg yolk lecithin (∼72% L-α-phosphatidylcholine) was purchased from Fisher Chemical Co., Loughborough, England. Silymarin (SIL) was supplied by MADAUS (Legalon®, Koeln, Germany). TAA and Pluronic F127 (poloxamer 407) (P-F127) were obtained from Sigma‒Aldrich Chemicals Co. (St. Louis, MO, U.S.A.). As a common vegetable, purslane was purchased from Al-Naqiti Herbs Company, Mansoura, Egypt. Methyl alcohol and chloroform were from El-Nasr Company for Pharmaceutical Chemicals, Abu-Zaabal, Cairo, Egypt. Each assay included a description of the kit/reagent supplier. All further chemicals were of high analytical quality.

### Methods

#### Purslane seed oil extract preparation

The extract was prepared at NAWAH Scientific Labs, Al-Mokattam, Cairo, Egypt. The plant stalks, stones, and seeds were cleaned manually and dried. The plant seeds were subsequently subjected to direct sunlight. Then, the purslane seeds were oven-dried for 12 h at 50°C, and the powder was made with a grinder. The prepared powder with a particle size of 0.5–1 mm was kept in a vacuum dryer until use. Ground samples of 3125 g were mixed with 6 L of methanol and then homogenized for 30 min via a T 50 digital Ultra-Turrax^®^ (IKA Labortechnik, Germany), followed by another 90 min of homogenization after adding chloroform-methanol mixture (at a ratio of 2:1). Later, the extract was filtered through a Buchner funnel, and the precipitate was washed with an additional volume of the 2:1 a chloroform–methanol mixture. The lipid layer was collected and then dried using a rotary evaporator under vacuum at 40°C, producing a brown residue weighing 138.125 g [25,26]. The oil yield from chloroform-methanol extraction is higher than that obtained by other extraction methods [25].

#### Gas chromatography-mass spectrometry analysis of purslane seed oil extract

The GC-MS-based identification of the purslane seed oil extract (PSE) phytochemicals implied the use of GC-MS. The extract had to be derivatized first to facilitate the volatility improvement as well as thermal stability of the compounds. Essentially, the sample was mixed with sodium hydroxide, acidified with hydrochloric acid, then extracted with ethyl acetate. The organic layer after the experiment was blown to dryness under the stream of nitrogen gas at 40°C. The dry precipitate was then derivatized by dissolving the precipitate in 50 μl of bis(trimethylsilyl)trifluoroacetamide that contained 1% trimethylchlorosilane as well as 50 μl of pyridine, converting the functional groups such as the -OH and -COOH to the trimethylsilyl (TMS) derivatives. The GC-MS analysis was performed using the Agilent Technologies system (7890B GC with a 5977A MSD) equipped with an HP-5MS capillary column (30 m length × 0.25 mm internal diameter × 0.25 μm film thickness) following standard protocols for seed oil profiling [27], with slight modifications in carrier gas and oven temperature program. The hydrogen as the carrier gas maintained a steady flow of 1.1 ml/min. A 1.0 μl sample was introduced in splitless mode. The temperature program of the oven had the beginning temperature at 40°C (held for 1 min), ramped to 200°C at a temperature ramp of 10°C/min (held for 1 min), then to 220°C at a temperature ramp of 20°C/min (held for 1 min), then to 300°C at a temperature ramp of 30°C/min (held for 3 min). The temperatures of the ion source as well as the injector were maintained at 300°C and 230°C, respectively. Mass spectra were acquired in electron ionization mode at 70 eV, with a scan range of m/z 50–600. Compound identification was achieved by comparing the mass spectrum fragmentation patterns and retention times of the sample constituents with those of reference compounds available in the Wiley and NIST Mass Spectral libraries.

#### Preparation of PSE-PMLs

The Bangham method, which is commonly known as the thin film hydration method [28], was used to prepare PSE-PMLs. Lecithin from egg yolk (600 mg), P-F127 (150 mg), and purslane seed oil extract (500 mg) were dissolved in a methanol:chloroform mixture (at a ratio of 3:2). The organic solvents were evaporated under vacuum for 30 min at 50°C and 150 revolutions per minute (rpm) until a thin film of lipids formed. The formed film was hydrated with 10 ml of distilled water for 45 min at the same temperature and rpm under normal pressure [29]. The formed PSE-PMLs were kept in the refrigerator for further evaluation.

##### Percentage entrapment efficiency measurement

The unentrapped extract was separated from the PSE-PMLs via centrifugation. 1 ml of the PSE-PMLs dispersion was centrifuged in an ultra-cooling centrifuge (Beckman, Fullerton, Canada) for 1 h at 22 000 rpm and 4°C [30]. The unentrapped extract in the separated supernatant was spectrophotometrically measured via a UV spectrophotometer (UV-1601 PC, Shimadzu, Kyoto, Japan) at a predetermined λ_max_ equal to 286 nm after being diluted with methanol. Percentage entrapment efficiency (EE%) was calculated via the following equation [31,32]:

$$\text{EE}\%=\left(\frac{{\text{Extract}}_{\text{Total}}-{\text{Extract}}_{\text{Supernatant}}}{{\text{Extract}}_{\text{Total}}}\right)\times 100.$$

##### Vesicular size, zeta potential, polydispersity index measurements

PSE-PMLs were evaluated for their vesicular size (VS), zeta potential (ZP), and polydispersity index (PDI) using a Malvern instrument (Model ZEN3600, Malvern Instrument Ltd., Worcestershire, U.K.) by diluting 3 drops of PSE-PMLs in 20 ml of distilled water at 25°C. Generally, VS is determined by the amount of light scattered from the vesicular surface, and PDI is calculated from the square of the light-scattering polydispersity [33,34]. ZP measurements rely on the electrostatic interaction between charged vesicular surfaces in an aqueous medium [35].

##### Morphological examination of PSE-PMLs

Transmission electron microscopy (TEM) (Jem-2100, JEOL, Tokyo, Japan) was utilized to perform morphological examination of the PSE-PMLs. The PSE-PMLs dispersion (0.1 ml) was diluted to 10 ml using distilled water. One drop of the diluted dispersion was placed on a grid coated with carbon. The sample was left to dry in the air for 10 min, and then, unstained PSE-PMLs were imaged at a suitable magnification. TEM was operated at 80 kV at 25°C [36,37].

#### Animals

Adult male Wistar albino rats from the animal house of the National Research Center, Cairo, Egypt, weighing 100 g ± 20, were maintained in an environmentally controlled room with a relative humidity of 35%–375%, a 12 h light/dark cycle, and free access to food and water *ad libitum*. Rats were permitted to acclimatize to the conditions above for one week before the experiment. The animals were handled in accordance with the Guidance on the operation of the Animals (Scientific Procedures) Act 1986 and associated guidelines, the Principles of the NIH Guide for the Care and Use of Laboratory Animals (NIH Publication No. 86-23). All protocols concerning animal use were approved by the Research Ethics Committee for Animal Experimentation of the Faculty of Pharmacy, Cairo University (approval no. BC 3701). All efforts were made to minimize animal discomfort and suffering.

#### Experimental design

In our study, 24 rats were randomly separated into 4 groups (*n* = 6/group): the normal control (NC), TAA-induced ALF (TAA), SIL, and PSE-PMLs groups. The NC group received normal saline (1 ml/100 g) orally, whereas the SIL- and PSE-PMLs-pretreated groups were orally administered SIL (200 mg/kg) suspended in distilled water as a standard hepatoprotective agent and PSE-PMLs (500 mg/kg), respectively. The doses of SIL and PSE-PMLs were chosen based on studies on their hepatoprotective effects in rodent acute hepatotoxicity models [11,38–40]. Following pretreatment with normal saline, SIL, or PSE-PMLs for 7 successive days, all groups, except the NC group, were injected with TAA (350 mg/kg) [41] intraperitoneally to establish the acute liver injury animal model.

Twenty-four hours after TAA injection, blood samples were obtained from the retro-orbital plexus under mild diethyl ether anesthesia (Supplementary Figure S1). The rats were subsequently killed via spinal dislocation while they were in a fasting state, and their livers were collected. Two sets of experiments were performed for each group: one (*n* = 6) for biochemical measurements and another (*n* = 3) for histopathological investigation.

#### Blood biochemical studies

The blood samples were incubated at room temperature for 1 h. Following centrifugation via a Hettich centrifuge (Germany) at 4000 rpm for 15 min at room temperature, the sera were isolated for biochemical estimations. The clear, non-hemolyzed supernatants were rapidly removed, divided into four portions for each rat, and then stored at −80°C for subsequent analysis. Serum levels of alanine aminotransferase (ALT), aspartate aminotransferase (AST), and total and direct bilirubin were measured via quantitative colorimetric assay kits (catalog #A524150 and catalog #A559150; Teco Diagnostics, Anaheim, CA, U.S.A., and catalog #DIBR-180; BioAssay Systems, LLC, CA, U.S.A., respectively) per the manufacturer’s instructions.

The concentrations of inflammatory biomarkers, namely, interleukin-4 (IL-4) (catalog #MBS355442; MyBiosource, Inc., San Diego, CA, U.S.A.) and interferon gamma (IFN-γ) (catalog #CEK1602; Cohesion Bioscience, London, U.K.), were determined via enzyme-linked immunosorbent assay (ELISA) kits following the manufacturer's instructions. A standard curve was used to convert the optical density (OD) reads and calculate the amounts of IL-4 and IFN-γ in the samples.

#### Preparation of liver homogenate

Livers were quickly excised and homogenized in cold phosphate-buffered saline (10% w/v) via a Teflon homogenizer (Glas-Col., Terre Haute, U.S.A.). The homogenates were centrifuged at 4°C and 12000 rpm for 10 min to obtain the supernatants, and these samples were also stored at −80°C for subsequent determination.

The hepatic content of the following markers was measured in liver homogenates via relevant rat-specific ELISA kits according to the manufacturer’s protocol for the reagent kits: malondialdehyde (MDA), superoxide dismutase (SOD) activity, peroxisome proliferator-activated receptor-gamma coactivator 1-alpha (PGC1-α), p-JAK2, p-STAT3, tumor protein (p53), B-cell lymphoma 2 (BCL2), and active cysteine-aspartic acid protease 3 (caspase-3) (catalog #MBS268427, catalog #MBS036924, catalog #MBS1600213, catalog #MBS7269637, catalog #MBS9501569, catalog #MBS723886, catalog #MBS704330, and catalog #MBS7244630, respectively; MyBiosource, Inc., San Diego, CA, U.S.A.) as well as PPARγ (catalog #LS-F4266; LifeSpan BioSciences, Inc., Washington, U.S.A.).

#### Histopathology

The liver tissues were fixed in 10% neutral-buffered formalin for a day and dehydrated by passage through different grades of alcohol. The dehydrated liver tissue was cleared by immersion in xylene and then embedded in paraffin wax. Following deparaffinization and rehydration, 5 µm-thick slices were stained with hematoxylin‒eosin (H&E) to analyze the overall histological structure. Liver sections were observed under a BX 51 light microscope (Olympus, Tokyo, Japan). The samples were inspected by an experienced pathologist in a blinded manner to avoid bias. Additionally, the degree of liver damage was semi-quantitatively graded as follows: (−) indicates absence (0%–25%), (+) indicates mild severity (25%–50%), (++) indicates moderate changes (50%–75%), and (+++) indicates marked severity (75%–100%).

#### Statistics

All the results are expressed as the mean ± standard error of mean (SEM), except for the characterization of the PSE-PMLs, where the measurements were carried out in triplicate and the results are presented as the mean ± standard deviations (SD). Using the Shapiro–Wilk test, we determined whether the results were normally distributed. Multiple comparisons between groups were achieved via one-way ANOVA followed by post hoc analysis (Tukey). The statistical test used is described in the figure legends. Pearson’s correlation was used to assess the relationships between the studied variables. The significance level was set at *P <*0.05. Statistical analyses were performed via Graph Pad Prism software version 8.4.2 (San Diego, CA, U.S.A.).

## Results

### Phytochemical characterization of PSE by GC-MS

**Figure 1:**
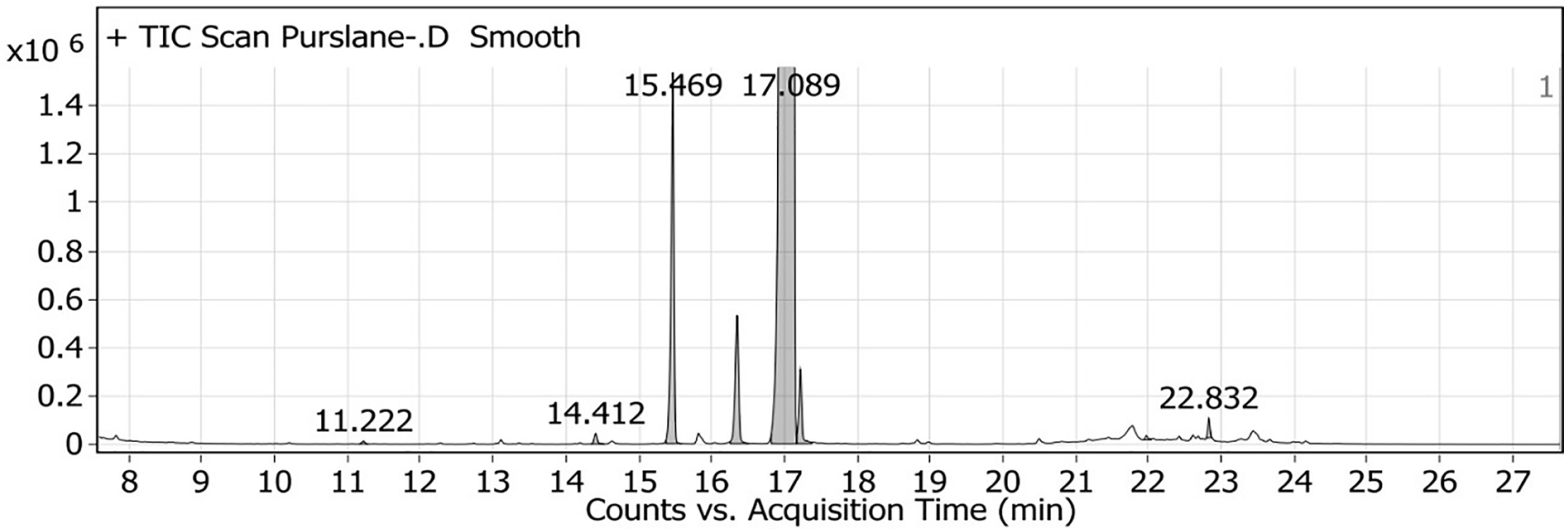
Representative GC-MS chromatogram (zoomed scale) of PSE showing α-linolenic acid as the predominant peak (RT = 17.089 min) along with minor peaks at RTs = 11.222, 14.412, 15.469, and 22.832 min The major peak was intentionally truncated due to axis scaling to highlight the minor constituents.

GC-MS analysis was employed to define the composition of the PSE phytochemicals, which is crucial to the disclosure of their bioactivity. The identified compounds are summarized in Table 1, which revealed the presence of several bioactive constituents. The most abundant compound was α-linolenic acid (an omega-3 fatty acid), which constituted 98.68% of the total identified area. Based on the GC-MS analysis, the delivered dose of α-linolenic acid in the 500 mg/kg PSE-PMLs was approximately 494 mg α-linolenic acid/kg body weight. Other notable compounds included palmitic acid (0.78%), caffeic acid (0.31%), and stearic acid (0.16%). Furthermore, important minor constituents were identified, such as γ-tocopherol (a form of vitamin E) and β-sitosterol (a phytosterol), alongside phenolic acids like 4-hydroxybenzoic acid and 4-coumaric acid. This rich profile of unsaturated fatty acids, phytosterols, tocopherols, and phenolic compounds is consistent with the reported antioxidant, anti-inflammatory, and hepatoprotective properties of purslane seed oil (Figure 1).

**Table 1:** Bioactive compounds identified in purslane seed oil extract using GC-MS analysis

Peak	Retention time (min)	Compound name	Molecular formula	Area	Area sum%
1	11.222	4-Hydroxybenzoic acid, 2TMS derivative	C_13_H_22_O_3_Si_2_	33971.86	0.01
2	14.412	4-Coumaric acid, 2TMS	C_15_H_24_O_3_Si_2_	126838.96	0.02
3	15.469	Palmitic acid, TMS derivative	C_19_H_40_O_2_Si	4480394.2	0.78
4	16.351	Caffeic acid, 3TMS derivative	C_18_H_32_O_4_Si_3_	1793315.62	0.31
5	17.089	α-Linolenic acid, TMS derivative^*^	C_21_H_38_O_2_Si	568935241	98.68
6	17.22	Stearic acid, TMS derivative	C_21_H_44_O_2_Si	926627.87	0.16
7	21.975	γ-Tocopherol, TMS derivative	C_31_H_56_O_2_Si	52773.68	0.01
8	22.832	β-Sitosterol, TMS derivative	C_32_H_58_OSi	171353.95	0.03

* α-linolenic acid was the predominant compound (∼98% of total detected area).

### Characterization of PSE-PMLs

**Figure 2:**
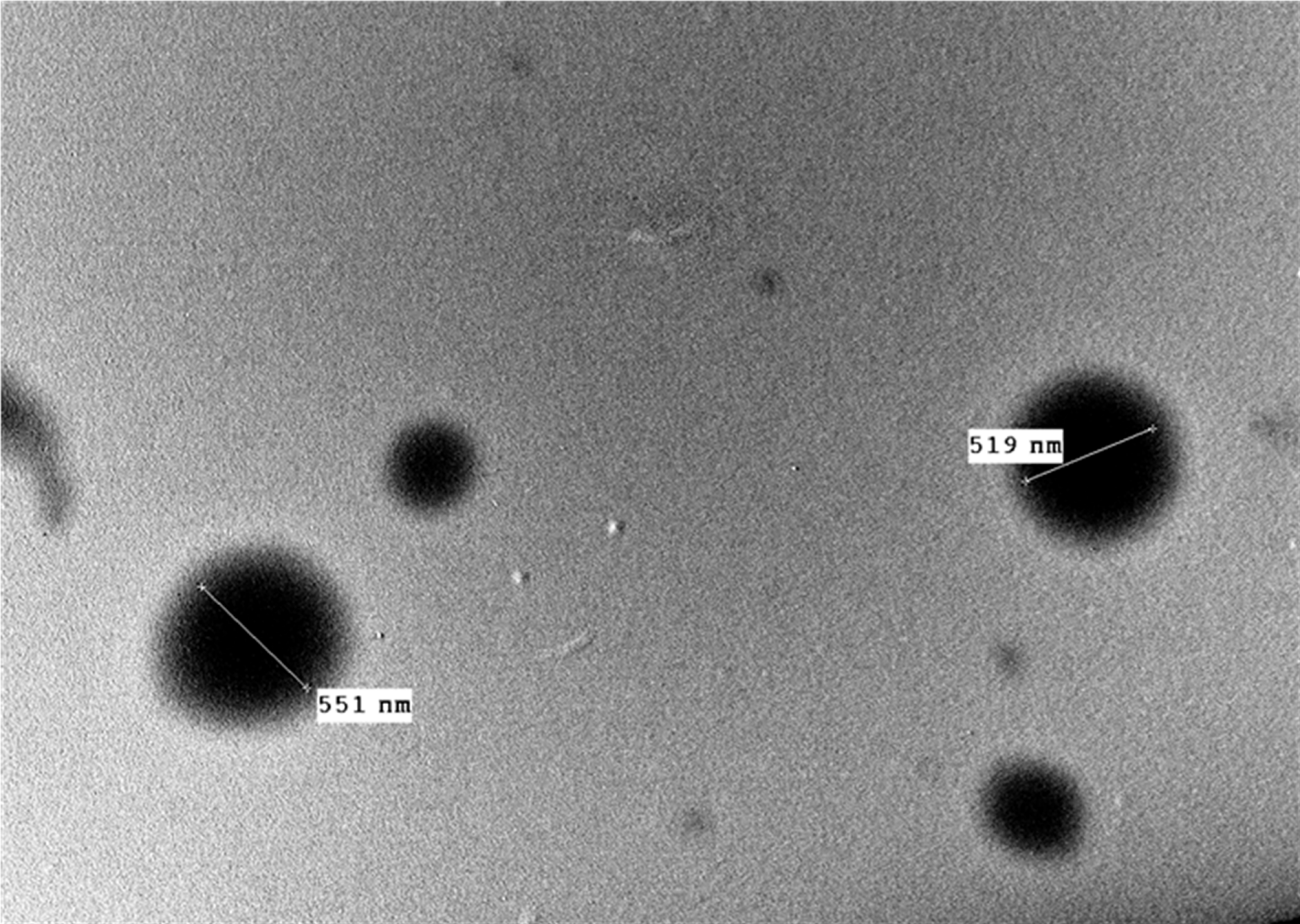
Morphological examination of PSE-PMLs PSE-PMLs, purslane seed extract-Pluronic-modulated liposomes.

EE is a vital parameter for NDCs. The EE of the PSE-PMLs was 87.8% ± 0.17. The VS of the PSE-PMLs was found to be 477.2 nm ± 8.76. The ZP and PDI of the PSE-PMLs were found to be −33.1 mV ± 1.62 and 0.53 ± 0.04, respectively. With respect to the morphological examination of the PSE-PMLs displayed in Figure 2, the PSE-PMLs showed properly formed spheres with VSs comparable to those detected via a Zetasizer.

### PSE-PMLs reduced elevated liver function tests

**Figure 3:**
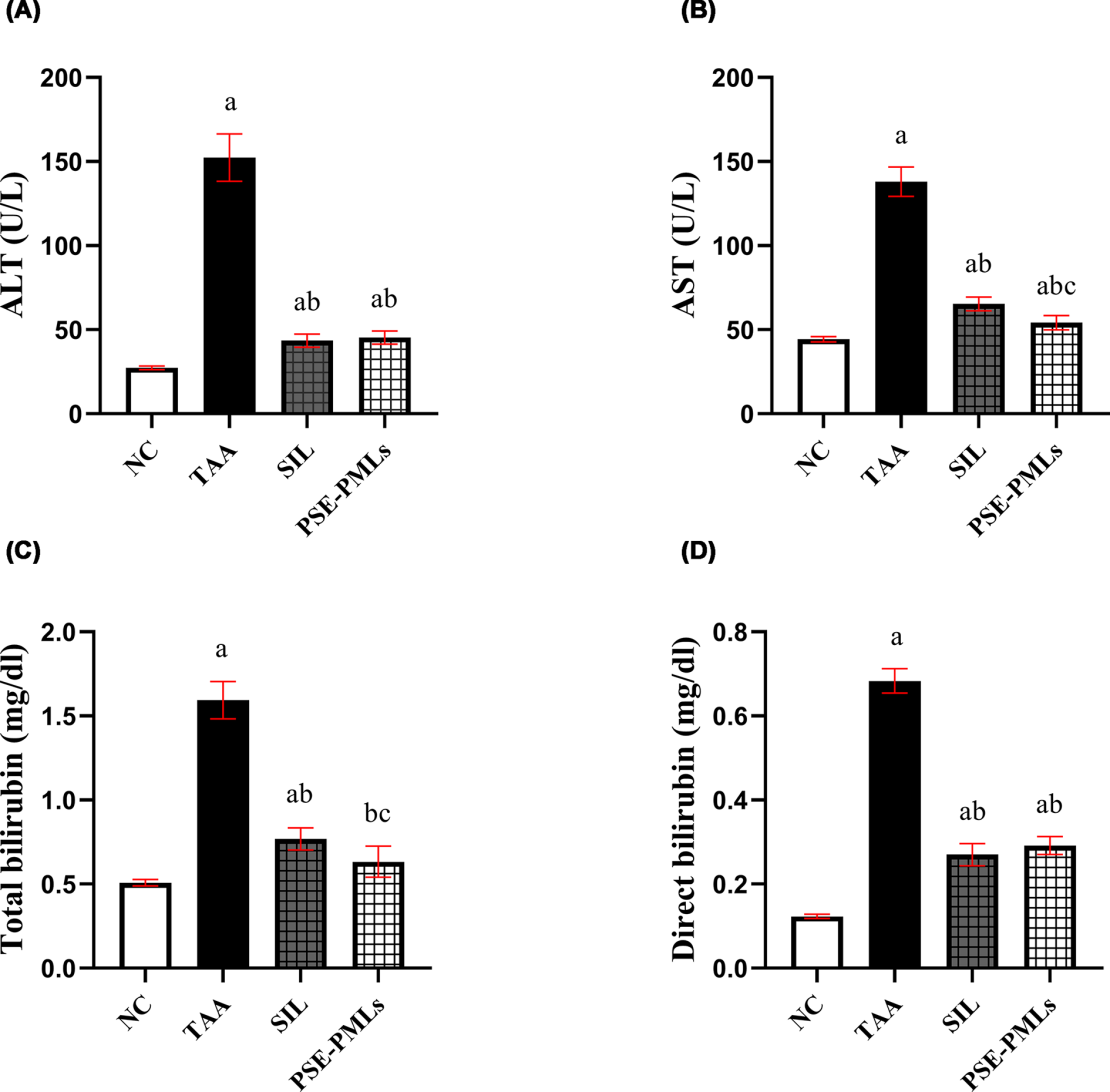
PSE-PMLs reduced elevated liver function tests. The data were analyzed via one-way ANOVA followed by the Tukey‒Kramer test, with each column with a vertical line representing the mean ± SEM. The letters (a, b, and c) represent significant differences from the NC group, TAA group, and SIL group, respectively. Significance was considered at *P* <0.05. ALT, alanine aminotransferase; AST, aspartate aminotransferase; PSE-PMLs, purslane seed extract-Pluronic-modulated liposomes. ALT (A), AST (B), total bilirubin (C) and direct bilirubin (D).

Compared with NC rats, TAA treatment markedly deteriorated liver function, as shown by elevated serum levels of the hepatic enzymes ALT and AST (457.8% and 211.5%, respectively) (*P* <0.0001). Notably, these levels were significantly lower in the SIL- and PSE-PMLs-pretreated groups (*P* <0.0001) than in the TAA group (Figure 3A,B). Moreover, PSE-PMLs afforded a significantly greater reduction in AST levels than did SIL.

Compared with the NC group, the TAA-treated group presented a highly significant increase in the serum total and direct bilirubin concentrations (*P* <0.0001). The serum total and direct bilirubin levels in the TAA-treated group subjected to SIL and PSE-PMLs were significantly lower than those in the TAA-treated group (*P* <0.0001) (Figure 3C,D). Notably, compared with SIL, PSE-PMLs significantly improved total bilirubin levels and markedly reduced total bilirubin levels to normal levels. These effects collectively indicate that PSE-PMLs may effectively ameliorate TAA-induced acute liver injury.

### PSE-PMLs attenuated TAA-induced liver oxidative stress and up-regulated antioxidant enzymatic activity

**Figure 4:**
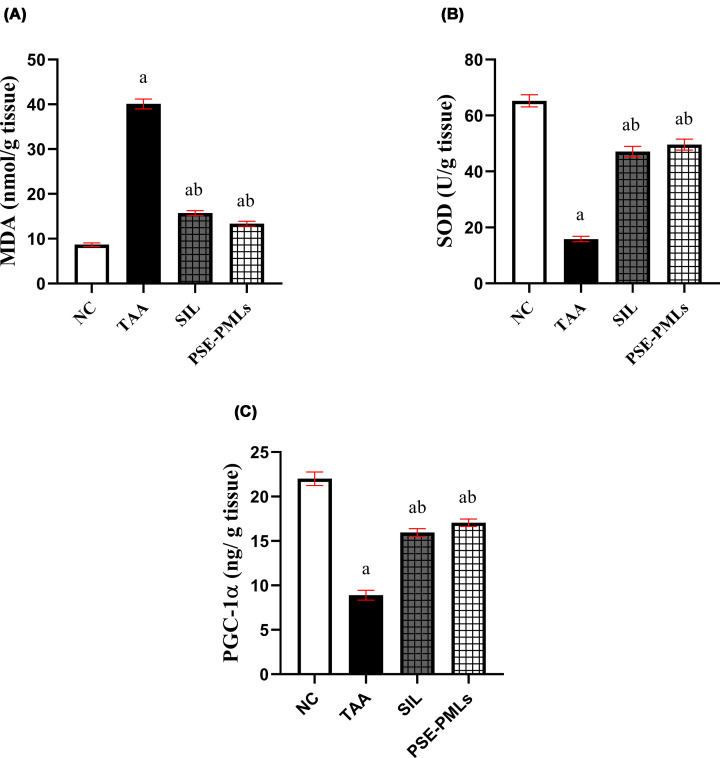
PSE-PMLs attenuated TAA-induced liver oxidative stress and up-regulated antioxidant enzymatic activity. The data were analyzed via one-way ANOVA followed by the Tukey‒Kramer test, with each column with a vertical line representing the mean ± SEM. The letters (a, b, and c) represent significant differences from the NC group, TAA group, and SIL group, respectively. Significance was considered at P <0.05. MDA, malondialdehyde; SOD, superoxide dismutase; PGC1α, peroxisome proliferator-activated receptor-gamma coactivator 1-alpha; PSE-PMLs, purslane seed extract-Pluronic-modulated liposomes. MDA (A), SOD (B) and PGC-1α (C).

Induction of ALF by TAA in rats could increase the reactive oxygen species (ROS) production and evoke oxidative stress conditions, progressively leading to liver dysfunction. The hepatic content of the oxidative stress biomarker MDA significantly increased in the TAA group (*P* <0.0001), which was effectively amended by SIL and PSE-PML pretreatments by 60.5% and 66.7%, respectively (*P* < 0.0001). In contrast, the levels of hepatic antioxidant enzyme, SOD activity, were significantly decreased (3.9-fold) in the TAA group compared with the NC group (*P* <0.0001). SIL and PSE-PMLs pretreatment significantly attenuated the decrease in SOD activity compared with that in the TAA group (*P* <0.0001) (Figure 4A,B).

As shown in Figure 4C, TAA administration markedly reduced the hepatic PGC1-α level by 59.5% compared with that in the NC group (*P* <0.0001). Furthermore, PSE-PMLs significantly elevated the PGC1-α level, which surpassed that in the TAA group by 92%. Similarly, the SIL group presented significantly elevated PGC1-α levels, with no significant difference from those of the PSE-PMLs group. Therefore, PSE-PMLs showed potent effects in modifying TAA-positive systemic antioxidant exhaustion.

### PSE-PMLs ameliorated TAA-induced liver inflammation

**Figure 5:**
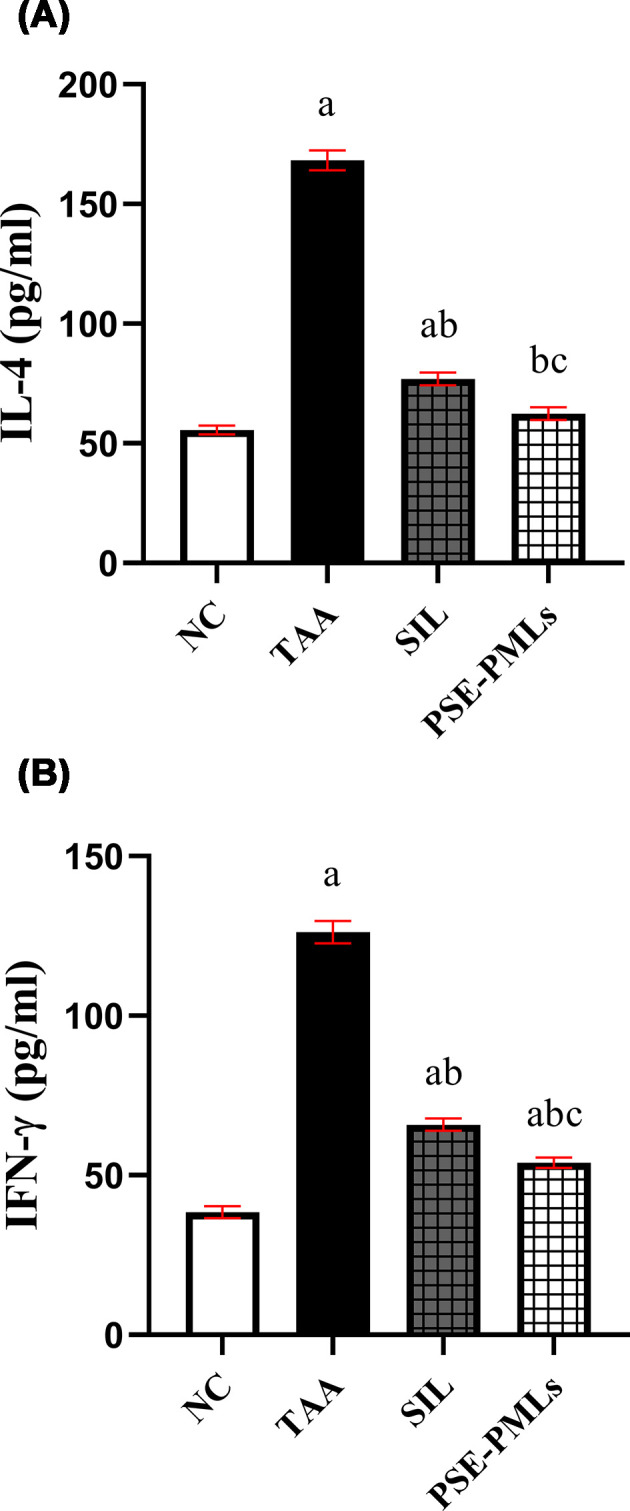
PSE-PMLs ameliorated TAA-induced liver inflammation. The data were analyzed via one-way ANOVA followed by the Tukey‒Kramer test, with each column with a vertical line representing the mean ± SEM. The letters (a, b, and c) represent significant differences from the NC group, TAA group, and SIL group, respectively. Significance was considered at P <0.05. IFN-γ, interferon gamma; IL-4, interleukin-4; PSE-PMLs, purslane seed extract-Pluronic-modulated liposomes. IL-4 (A) and IFN-γ (B).

The serum levels of the inflammatory mediators, IL-4 and IFN-γ, were significantly greater in the TAA group than in the NC group (*P* <0.0001) (Figure 5), indicating that TAA induced a severe liver inflammatory response. Compared with that in the TAA group, the increase in these inflammatory cytokines was significantly suppressed in the SIL group, by nearly 54% for IL-4 and 48% for IFN-γ. Promisingly, pretreatment with PSE-PMLs also significantly restrained the increased levels of IL-4 (*P* <0.0001), thereby restoring normal levels (*P* = 0.3889). Moreover, pretreatment with PSE-PMLs also caused a significant 2-fold reduction in the elevated IFN-γ serum levels in rats with ALF. The effect of PSE-PMLs on inflammatory markers was significantly greater than that of SIL (*P* = 0.0126 for IL-4 and *P* = 0.0093 for IFN-γ). These data suggest a protective effect of PSE-PMLs against the TAA-induced inflammatory response.

### PSE-PMLs interfered with the p-JAK2/p-STAT3/PPARγ pathway

**Figure 6:**
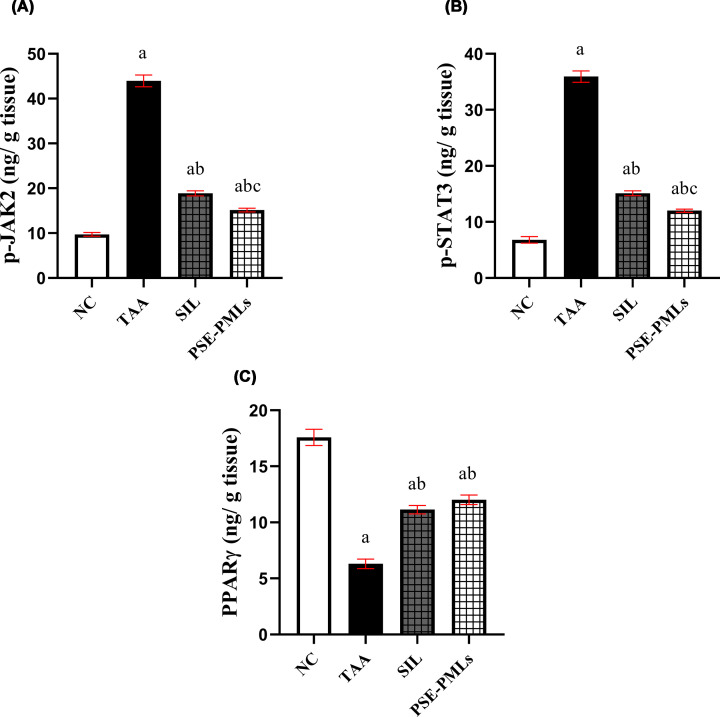
PSE-PMLs interfered with the p-JAK2/p-STAT3/PPARγ pathway. The data were analyzed via one-way ANOVA followed by the Tukey‒Kramer test, with each column with a vertical line representing the mean ± SEM. The letters (a, b, and c) represent significant differences from the NC group, TAA group, and SIL group, respectively. Significance was considered at *P* <0.05. p-JAK2, phosphorylated Janus kinase 2; PPARγ, peroxisome proliferator-activated receptor gamma; PSE-PMLs, purslane seed extract-Pluronic-modulated liposomes; p-STAT3, phosphorylated signal transducer and activator of transcription 3. p-JAK2 (A), p-STAT3 (B) and PPARγ (C).

We explored the effect of PSE-PML pretreatment on JAK2-STAT3 signaling, a crucial transcription factor involved in cytokine release in liver diseases. As shown in Figure 6A and B, the induction of ALF significantly increased the hepatic content of p-JAK2 and p-STAT3 in the TAA group by nearly 78% and 81%, respectively, compared with the corresponding control values (*P* <0.0001). The SIL group presented significantly decreased hepatic levels of p-JAK2 and p-STAT3 (*P* <0.0001). However, compared with the TAA group, PSE-PML pretreatment significantly caused a three-fold down-regulation in both p-JAK2 and p-STAT3 hepatic levels, with significant differences from the SIL group (*P* = 0.0131 for p-JAK2 and *P* = 0.0133 for p-STAT3).

The results also indicated that the induction of ALF with TAA significantly reduced the hepatic PPARγ level by 64%. On the other hand, SIL and PSE-PML pretreatments markedly increased the PPAR*γ* level in liver tissues by 43% and 47.5%, respectively (*P* <0.0001), compared with the TAA level. However, the hepatic PPAR*γ* level did not significantly differ between the two groups (Figure 6C).

### PSE-PMLs diminished hepatocyte apoptosis

**Figure 7:**
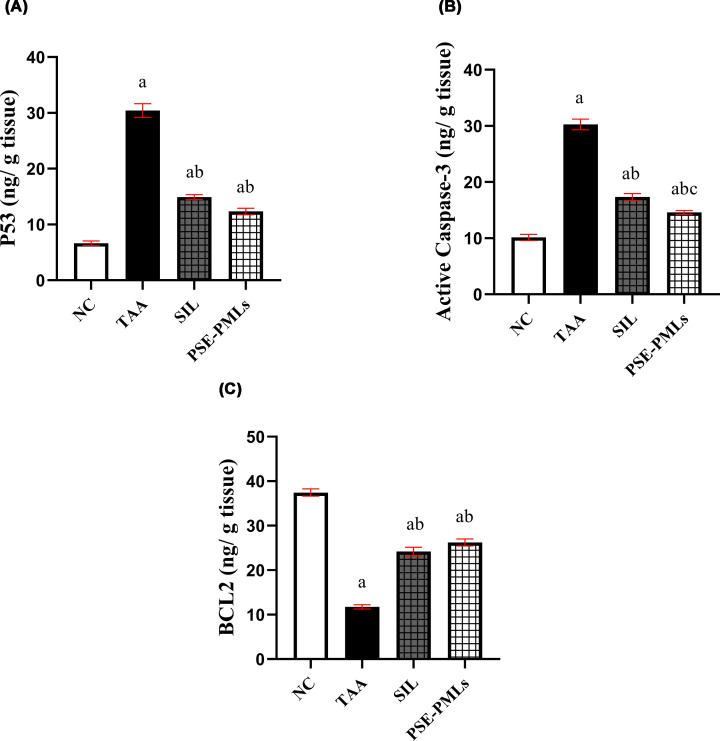
PSE-PMLs inhibited hepatocyte apoptosis. The data were analyzed via one-way ANOVA followed by the Tukey‒Kramer test, with each column with a vertical line representing the mean ± SEM. The letters (a, b, and c) represent significant differences from the NC group, TAA group, and SIL group, respectively. Significance was considered at *P* <0.05. BCL2, B-cell lymphoma 2; caspase 3, cysteine-aspartic acid protease 3; p53, tumor protein p53; PSE-PMLs, purslane seed extract-Pluronic-modulated liposomes. P53 (A), Caspase-3 (B) and BCL2 (C).

The hepatic level of p53 in the TAA group was 4.6 times greater than that in the NC group (*P* <0.0001). However, the overproduction of p53 induced by TAA was reduced by 51% and 59% in the SIL- and PSE-PML-pretreated groups, respectively (*P* <0.0001) (Figure 7A). Compared with the NC, TAA caused a significant increase in hepatic active caspase-3 (66%) (*P* <0.0001). SIL and PSE-PML administration significantly reduced active caspase-3 compared with that in the TAA group by 42.9% and 52%, respectively (*P* <0.0001), with PSE-PMLs being more potent than SIL in this respect (*P* = 0.0333) (Figure 7B).

Our results also revealed that, compared with that in the TAA group, the degree of BCL2 expression tended to increase significantly in the SIL (51.4%) and PSE-PMLs (55.1%) pretreatment groups. No significant variations in the hepatic level of the antiapoptotic protein BCL2 were observed between the SIL and PSE-PML pretreatment groups (Figure 7C). Taken together, these findings indicate that PSE-PMLs may also exert antiapoptotic impacts on the livers of TAA-treated rats.

### PSE-PMLs mitigated histopathological changes in liver tissues

**Figure 8:**
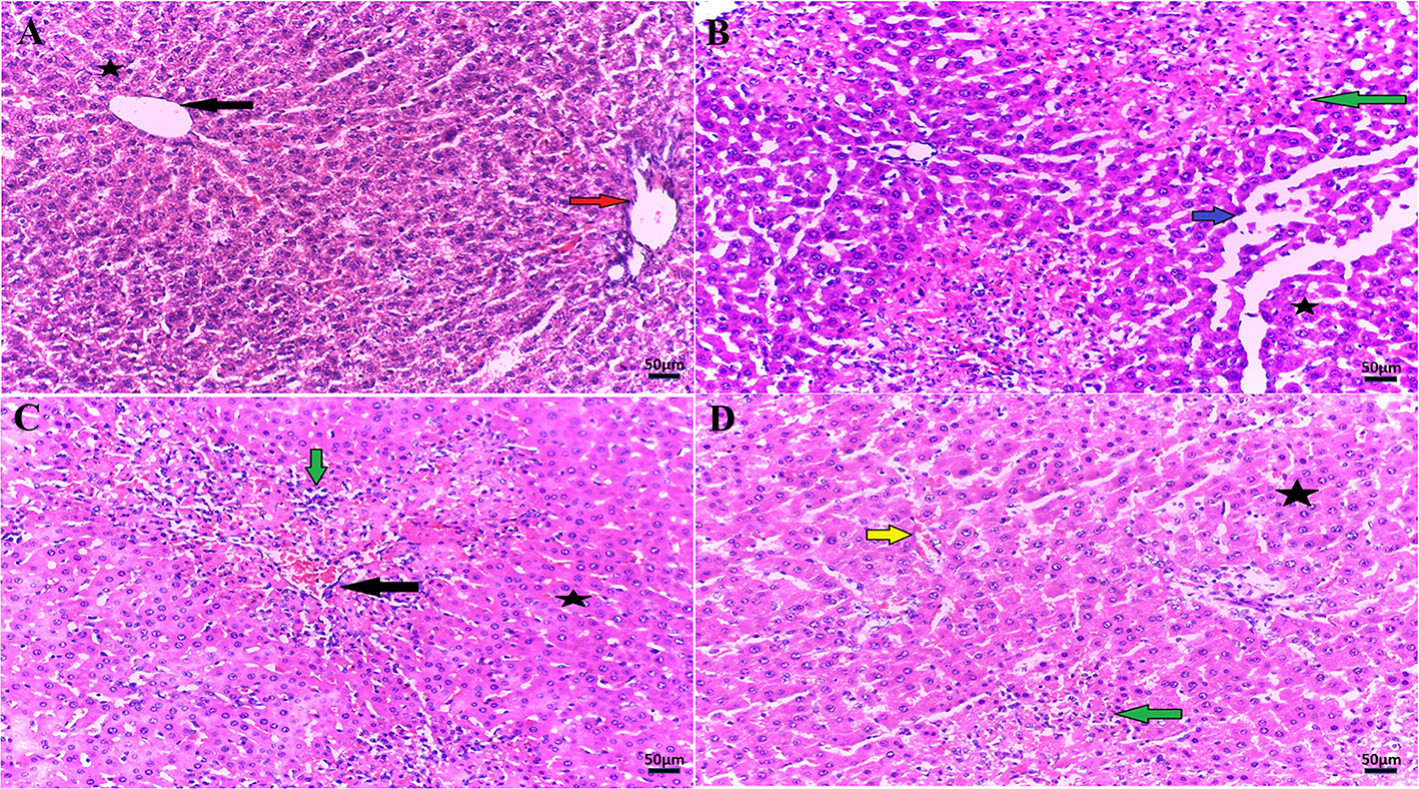
PSE-PMLs mitigated histopathological changes in liver tissues Representative photomicrographs of H&E-stained livers (200×) showing no histopathological changes: normal central vein (black arrow), normal portal tract (red arrow), and normal hepatocytes (star) in the NC group (**A**); degenerated hepatocytes (star), intercellular edema (blue arrow), and multiple necroinflammatory foci (green arrow) in the TAA group (**B**); moderately congested central vein (black arrow) and multiple inflammatory cells (green arrow) and degenerated with some normal hepatocytes (star) in the SIL group (**C**); apparently normal hepatocytes (star) and slightly congested sinusoids (yellow arrow) with little inflammatory cell infiltration (green arrow) in the PSE-PMLs group (**D**).

As shown in Supplementary Figure S2 and Figure 8, the hepatocytes in the NC group exhibited a normal lobular architecture, a normal central vein, and clear hepatic cords extended outward from the central vein, whereas the normal architecture of hepatocytes disappeared in the TAA group, accompanied by severe portal vein congestion, intercellular swelling, necrosis, and severe inflammatory cell infiltration, demonstrating the successful induction of the acute hepatic injury model. The SIL group samples presented moderate multifocal inflammation, degenerated hepatocytes still present, and a reduced necrotic mass. Alternatively, PSE-PMLs pretreatment resulted in significant improvement, and apparently normal liver sections were observed in almost all the examined rats except for a few sections that presented slightly congested vessels and few necro inflammatory foci. These observations were mirrored on hepatic injury scores (Table 2) where high injury records were apparent in TAA group, while they were obviously improved by SIL pretreatment and almost reversed by PSE-PMLs pretreatment.

**Table 2:** Effect of PSE-PMLs on the score of hepatic histopathological changes in TAA-induced-ALF in rat

Groups	Histopathological Alterations	Central vein congestion	Inflammatory cell infiltrates	Degenerative/necrotic changes
**NC group**		−	−	−
**TAA group**		+++	+++	+++
**SIL group**		+	++	+
**PSE-PMLs group**		−	+	−

(−) indicates absence (0%–25%), (+) indicates mild severity (25%–50%), (++) indicates moderate changes (50%–75%), and (+++) indicates marked severity (75%–100%). *Abbreviations: NC, normal control; PSE-PMLs, purslane seed extract-Pluronic-modulated liposomes; SIL, silymarin; TAA, thioacetamide.*

### Correlation analysis

**Figure 9:**
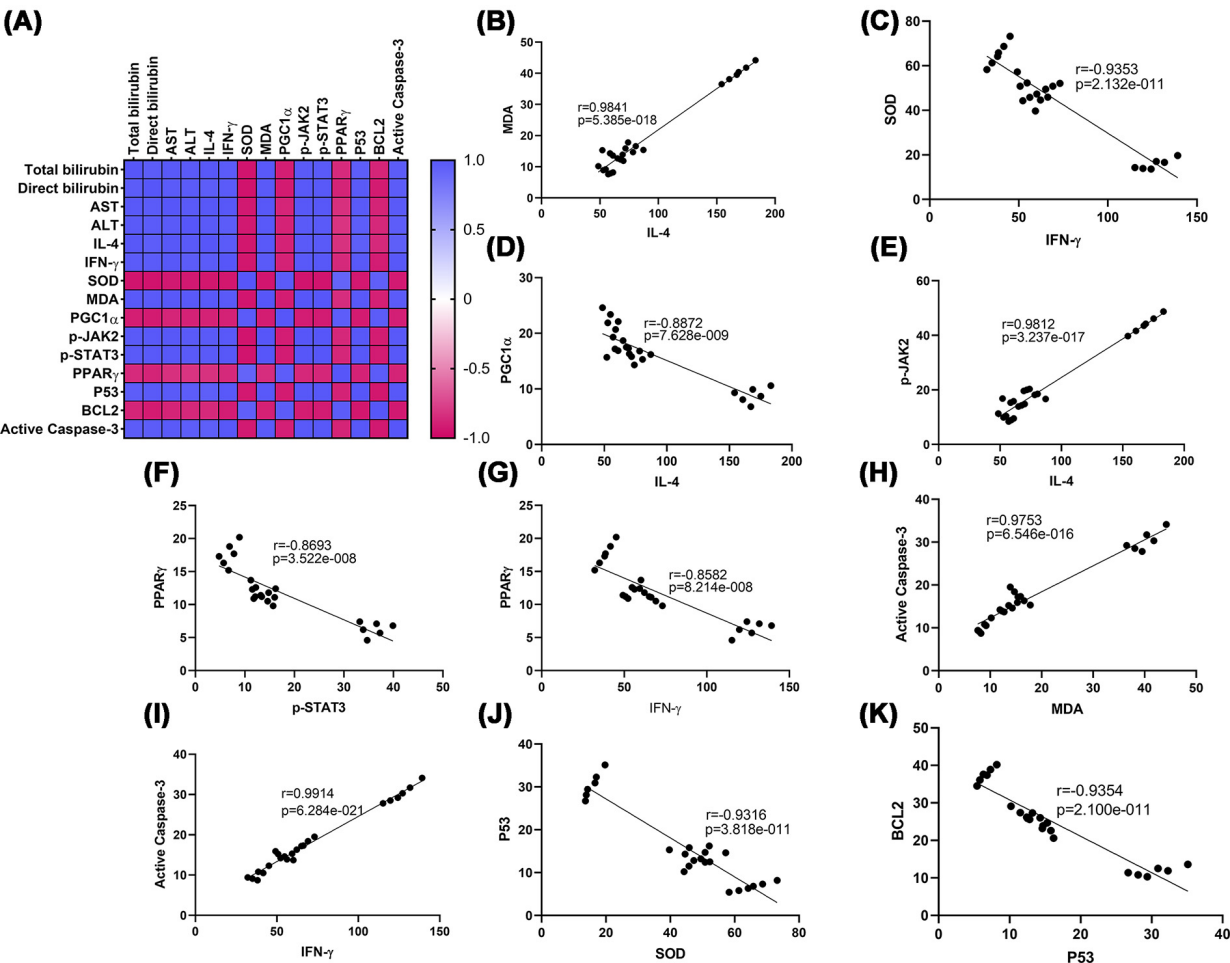
Heatmap of correlations, linear regression, and representative correlation analyses Heatmap of Pearson’s correlation matrix (**A**), correlation of MDA with IL-4 (**B**), correlation of SOD with IFN-γ (**C**), correlation of PGC1α with IL-4 (**D**), correlation of p-JAK2 with IL-4 (**E**), correlation of PPARγ with p-STAT3 (**F**), correlation of PPARγ with IFN-γ (**G**), correlation of active caspase-3 with MDA (**H**), correlation of active caspase-3 with IFN-γ (**I**), correlation of P53 with SOD (**J**), and correlation of BCL2 with P53 (**K**). Pearson’s correlation coefficient (*r*) and *P*-value are shown. In the heatmap, parameters are clustered via Pearson’s correlation as the distance metric. BCL2, B-cell lymphoma 2; caspase 3, cysteine-aspartic acid protease 3; IFN-γ, interferon gamma; IL-4, interleukin-4; MDA, malondialdehyde; p-JAK2, phosphorylated Janus kinase 2; PGC1α, peroxisome proliferator-activated receptor-gamma coactivator 1-alpha; PPARγ, peroxisome proliferator-activated receptor gamma; p-STAT3, phosphorylated signal transducer and activator of transcription 3; SOD, superoxide dismutase; p53, tumor protein p53.

Pearson’s correlation matrix revealed significant linear correlations between liver functions, inflammatory markers and their molecular regulators, and oxidative stress and apoptotic markers, as shown in Supplementary Table S1. Pearson’s correlation analysis revealed strong negative associations between the inflammatory mediators (IL-4 and IFN-γ), with the following parameters: SOD, PGC1-α, and PPARγ. Whereas MDA was positively correlated with inflammatory and apoptotic markers. Moreover, p-JAK-2 and p-STAT-3 were significantly associated with the inflammatory markers and were negatively correlated with PPARγ. However, p53 and caspase-3 were positively correlated with inflammatory and oxidative stress mediators and negatively associated with the antiapoptotic protein BCL2 (Figure 9).

## Discussion

ALF is a life-threatening condition that can rapidly lead to multi-organ failure and death. Despite advancements in the development of novel treatment modalities, ALF is still associated with high mortality rates. TAA has been proven to have an obvious liver-damaging effect and has been used for years as a classic hepatotoxin in experimental liver injury models. The clinical features as well as biochemical and histopathological alterations associated with TAA-induced liver damage have been widely studied and closely resemble those associated with acute liver damage in humans. The pathological high dose of TAA is known to induce oxidative stress along with a decrease in antioxidant status, inflammatory cell infiltration, and apoptosis, ultimately resulting in liver failure [42–45]. Unsurprisingly, the protection of the liver against toxic injuries remains a major clinical challenge. The present study is the first to examine the potential hepatoprotective effect of PSE-PMLs against TAA-induced acute liver toxicity in rats.

In the present study, TAA injection caused an increase in serum levels of liver function tests and inflammatory cytokines (IL-4 and IFN-γ). In addition, the present study revealed that oxidative stress plays a crucial role in the pathophysiology of TAA-induced acute liver injury in rats, as demonstrated by an increase in the lipid peroxidation byproduct MDA and a decrease in the intracellular antioxidant SOD activity in liver tissues. These findings were consistent with the histopathological alterations induced by TAA. Overall, these findings confirm that the administration of TAA triggered hepatocellular damage and liver dysfunction.

On the other hand, the pretreatment of rats with PSE-PMLs resulted in the maintenance of the histological integrity of hepatocytes, which is associated with a significant reversal of the TAA-induced increase in the serum levels of liver biochemical markers. Purslane has demonstrated hepatoprotective effects against liver damage and fibrosis caused by numerous toxins. It has the ability to normalize all disturbed biochemical parameters [11]. The protective effect of purslane extract against carbon tetrachloride-induced hepatic toxicity was previously investigated, and both markers of liver function and liver histopathological alterations improved after purslane treatment, indicating its potential to protect the liver from injury [46].

Oxidative stress is strictly correlated with the inflammatory response. Overproduction of ROS diminishes intracellular antioxidant defense mechanisms, causing oxidative stress and pro-inflammatory mediator release, which exacerbates inflammation and may even worsen liver injury [47]. TAA-mediated hepatotoxicity may enhance Kupffer cell activity, leading to elevated levels of oxidative stress, inflammation, and hepatocellular necrosis [48]. In contrast, purslane treatment has been previously shown to be associated with decreased levels of the oxidative damage marker MDA and increased levels of the antioxidant enzyme SOD in the liver, kidney, heart, and testes of rats, suggesting its protective effects against oxidative stress, as evidenced herein [49]. Purslane’s antioxidant effects may be because of its comparatively high concentration of the vitamins C and E, which are known antioxidants [50].

A number of conditions can cause inflammation of the liver, such as drug-induced liver injury, nonalcoholic steatohepatitis, and viral infections. This condition is characterized by a pro-inflammatory cascade triggered by the hepatocyte death, wherein immune cells respond to the cytokines released from hepatocytes that are moribund. The extent of activation of the immune system can impact the severity of the damage, as monocytes and neutrophils are rapidly recruited to the site of injury and can either exacerbate ongoing tissue damage or facilitate the healing of liver injury [51].

IL-4 has a pro-inflammatory role in the development of hepatitis in drug-induced hepatotoxicity. However, the role of IL-4 in liver injury remains controversial, as it has also been suggested to play a protective role during disease progression. It is a complex cytokine whose role in autoimmunity varies between anti- and pro-inflammatory. In experimental drug-induced liver injury, where hepatotoxicity mechanisms are being investigated, IL-4 is clearly pro-inflammatory; however, it plays an immunoregulatory role in response to autoantigens [52]. In support of our findings, it has been demonstrated that IL-4-deficient mice are resistant to hepatic injury following acetaminophen-induced hepatotoxicity [53].

In addition, IFN-γ promotes drug-induced hepatotoxicity and has previously been linked to hepatotoxicity in drug-overdosed patients. It performs pleiotropic functions in host defense against pathogens and tissue injury in autoimmune or inflammatory diseases, such as liver hepatitis [54]. In line with our findings, a previous study in a mouse model of acetaminophen-induced liver injury found that ablation of the IFN-γ gene reduced hepatotoxicity and hepatic inflammation, confirming the biological significance of this cytokine in drug-induced hepatic damage [55]. IL-4 and IFN-γ levels were shown to be linked to oxidative stress, as evidenced by their positive correlation with MDA and negative correlation with SOD in our study. Conversely, in harmony with our findings, purslane showed anti-inflammatory effects through the reduction of inflammatory mediators, including IL-4 and IFN-γ in various inflammatory and immune diseases [49]. Thus, PSE-PMLs may be potentially valuable in treating disorders related to inflammation and oxidant/antioxidant status and immune system imbalances.

Excessive pro-inflammatory cytokine release is a central trigger for hepatocyte damage. During inflammation, cytokine signal transduction is mainly mediated through the JAK/STAT signaling pathway [47]. JAK2 and STAT3 are among the most important families of protein tyrosine kinases accompanying uncontrolled cytokine release and liver inflammation. They are involved in the downstream events of cytokine receptor binding, which leads to the transcriptional activation of genes involved in inflammatory responses [23]. It has been reported that pro-inflammatory factors can activate and phosphorylate JAK2, which in turn induces STAT3 phosphorylation, thus participating in the regulation of gene transcription and mediating the expression of various pro-inflammatory factors [24]. In line, the present findings revealed a positive correlation between the inflammatory markers and the p-JAK2/p-STAT3 axis. TAA administration in the present study resulted in severe inflammatory response associated with significant increase in hepatic content of p-JAK2 and p-STAT3 along with decrease in hepatic PPARγ levels. Others have shown that STAT3 deficiency inhibits hepatocarcinogenesis and enhances biliary proliferation in TAA-induced liver injury [56], while JAK2/STAT5 signaling pathways are augmented in TAA-induced nephrotoxicity [57]. However, PPARγ acts as a regulator of JAK/STAT signaling, preventing their phosphorylation. Both PPARγ and JAK/STAT oppositely regulate each other [58,59]. Herein, both p-JAK2 and p-STAT3 were found to be negatively correlated with PPARγ, supporting these previous studies.

PPARγ is a nuclear receptor that assists in the regulation of many biological processes such as immunological responses, mitochondrial activity, energy metabolism, and antioxidant preservation. PPARγ plays a critical role in regulating the immune response by exerting an anti-inflammatory effect via the inhibition of macrophage and cytokine production [58,59]. Consistent with our findings, it has been reported that up-regulation of JAK/STAT and down-regulation of PPARγ results in inflammation and oxidative stress [58]. Interestingly, our results align with previous findings of the role of purslane extract in attenuating the development of drug-induced ulcerative colitis through the inhibition of the release of pro-inflammatory cytokines and the restoration of PPARγ levels, which were reduced due to the inflammatory reaction of the disease [60]. Moreover, in a mouse model of acute colitis, polysaccharide from purslane was able to down-regulate STAT3 and p-STAT3 levels [61].

PGC-1α is a transcriptional coactivator of PPARγ, which controls mitochondrial biogenesis and energy expenditure. Transcriptional coactivators typically manage extensive gene networks by stimulating gene expression through interactions with nuclear receptors [51,62]. PGC-1α is a powerful regulator of ROS metabolism in hepatocytes; ectopic expression of PGC-1α resulted in a reduction of MDA and an elevation of SOD, while PGC-1α down-regulation has the opposite effect [62]. Herein, PGC-1α was correlated negatively with MDA and positively with SOD. In addition, the inhibition of PGC-1α promotes hepatic inflammation in mice [63]. Consistently, in the present study, TAA administration caused a decrease in PGC-1α, which was found to be negatively correlated with IL-4 and IFN-γ; nonetheless, PSE-PML administration counteracted the decrease in PGC-1α.

TAA-induced liver apoptosis is thought to be primarily triggered by inflammation and oxidative stress [64]. The data in the present study revealed strong positive correlations between the inflammatory markers and oxidative stress indicators with the apoptotic enhancers. Many genes are involved in the apoptotic process. The activation of BAX (BCL2-associated X protein) enhances mitochondrial membrane permeability, triggers apoptosis, and counteracts the inhibitory influence of BCL2 on apoptosis. As well, intrinsic apoptosis is triggered by cytochrome C leakage, which is induced by caspase-3 activation [65]. Our results demonstrated that TAA caused a significant reduction in hepatic BCL2 content. This result ties well with the previous study wherein administration of an acute dose of TAA can cause hepatocyte apoptosis by decreasing the antiapoptotic protein BCL2 and increasing the expression of proapoptotic BAX and the apoptotic executioner caspase-3 [64]. Nevertheless, BCL2 has been documented to be upregulated by PPARγ, leading to mitochondrial stabilization and protection against apoptosis and oxidative stress [58].

Furthermore, nuclear p53 can significantly boost autophagy levels in cells via extrinsic apoptotic molecular pathways. It can also directly activate BAX, causing mitochondrial permeability and caspase activation, which leads to apoptosis [64]. P53 is a tumor suppressor phosphoprotein that helps with DNA repair by causing cell cycle arrest until repair is complete; however, if repair fails, it initiates apoptosis. As expected, the levels of p53 and caspase-3 were significantly increased in the livers of the TAA group and were negatively correlated with BCL2, as reported in previous studies [45,66,67]. Pretreatment with PSE-PMLs in our study countered apoptotic signals via decreasing caspase-3 activity and p53 hepatic content and further increasing the hepatic antiapoptotic BCL2 levels. In line with our findings, the expression of the caspase-3 gene has been reported to be down-regulated in the purslane-treated group in the context of induced brain injury in rats, which was attributed to increased expression of BCL2 [50]. In addition, the flavonoid content of purslane, particularly quercetin, inhibited the glucose oxidase-mediated DNA-binding activity of several sensitive transcription factors, including p53 [49].

The GC-MS profiling in the current study exposed a thunderous dominance of α-linolenic acid (∼98.68% of area detected), with minor yet bioactive components like caffeic acid, γ-tocopherol, β-sitosterol, 4-hydroxybenzoic acid, and 4-coumaric acid (Table 1). The supremacy of α-linolenic acid is consistent with prior findings on plant extracts as well as seed oils, which corroborates its pivotal role in hepatoprotection: α-linolenic acid itself has been found to improve hepatic steatosis and suppress inflammatory cytokine expression as well as control lipid metabolism through AMPK as well as PPAR pathways within liver models [68]. While α-linolenic acid dose (∼494 mg/kg) used in the present study exceeds those used in chronic models, this ‘pharmacological loading’ is essential for rapid intervention in ALF, which carries a higher mortality risk than gradual disease progression. Lower doses (60–100 mg/kg) effectively alleviate obesity-induced liver damage when administered chronically [69]. Similarly, daily supplementation of 165 mg/kg for 6 weeks is sufficient to enhance antioxidant defenses [70], and dietary α-linolenic acid effectively prevents chronic hepatic steatosis [71]. To counteract the acute apoptotic insult of LPS/D-GalN, a more potent dose was required to immediately inhibit the p-JAK2/p-STAT3 pathway and up-regulate PPAR-γ. This dose is mechanistically supported by studies where the high concentrations of α-linolenic acid reversed critical ER stress markers necessary for hepatocyte survival [72]. Thus, delivering this dose via a nano-liposomal system (PSE-PMLs) ensures superior bioavailability and protection against oxidation compared with conventional oil delivery, maximizing the prophylactic impact within the narrow clinical window of ALF.

On the other hand, caffeic acid is extensively documented to demonstrate hepatoprotection as well as antioxidant activities through the boosting of endogenous antioxidant systems (GSH and SOD) as well as repression of lipid peroxidation within liver damage models [73]. The co-presence of γ-tocopherol (a kind of the antioxidant vitamin E) adds yet another antioxidant dimension through the scavenging of lipid radicals as well as the stabilization of membrane lipids, whereas β-sitosterol possibly acts in the control of cholesterol metabolism as well as the repression of inflammatory signaling (e.g., through the PPARγ pathway). Therefore, the chemical profile revealed through GC-MS presents the mechanistic basis to substantiate our found decreased oxidative stress (reduced MDA and increased SOD) and down-regulation of the proinflammatory cytokines, as well as the repression of apoptosis within α-linolenic acid-based PSE-PML-pretreated animals.

In the present study, the elevated EE of PSE-PMLs is due to the lipophilic nature of the extract, as it prefers to stay in the lipophilic phase rather than in external aqueous media. The VS of PSE-PMLs is also satisfactory and appropriate for oral administration since it was reported that nanoparticles with sizes less than 500 nm can be easily absorbed from the gut. This promotes lymphatic uptake and increases the bioavailability of drugs by avoiding the first-pass effect [74]. PSE-PMLs were found to carry negative charges due to the phospholipid content of the lecithin [29]. The ZP value reflects the stability of the formed PSE-PMLs, as absolute ZP values ≥30 mV indicate the formation of physically stable dispersions with sufficient repulsion [75]. The PDI detected for PSE-PMLs was acceptable, as Cho et al*.* [76] reported that a suitable PDI ranges from 0.05 to 0.7. Although this PDI value (0.53) may reflect a relatively broad size distribution, it can be acceptable for* in vivo* applications without adversely affecting stability, targeting efficiency, or therapeutic performance [77]. Similar results were reported by Cunha et al. [77], as biogenically produced silver nanoparticles, with a PDI of 0.522, demonstrated effective *in vivo* wound-healing activity, indicating that their PDI did not hinder the biological performance. This can be attributed to the following points: first, the route of administration adopted here was the oral route, which offers flexibility regarding the particle size and size distribution of the administered formulations [78]. Second, some literature reported that PDI values up to 0.5 are regarded as tolerable for drug targeting and delivery applications [79]. Finally, the* in vivo* performance of NDCs does not solely depend on PDI but on the integration of all the formulation’s attributes, e.g., EE%, VS, and ZP. The shape of the NDCs affects cellular internalization, as it has been reported that nanoparticles with a spherical morphology such as PSE-PMLs were swiftly endocytosed [80].

The findings of the present study position PSE-PMLs as a sophisticated nano-prophylactic agent capable of modulating specific pro-inflammatory and apoptotic pathways in acute liver injury. The study also indicates that pretreatment with PSE-PMLs not only achieved comparable effects to those of silymarin in terms of ameliorating TAA-induced oxidative stress and increasing PPARγ, PGC1-α, and BCL2 levels but also achieved superior results in terms of improving liver function; decreasing inflammatory markers and apoptotic executioner caspase-3, as well as p-JAK2 and p-STAT3; and reversing histopathological changes.

## Conclusion

In conclusion, our findings suggest that PSE-PMLs can ameliorate TAA-induced acute liver inflammation and oxidative stress via the regulation of the p-JAK2/p-STAT3/PPARγ and apoptosis signaling axes. Therefore, PSE-PMLs may be a potential preventive option for protecting against acute liver diseases. The remarkable bioactivity of PSE-PMLs can be attributed to the synergistic effect of its rich phytochemical composition, predominantly α-linolenic acid, alongside other bioactive sterols, tocopherols, and phenolic compounds, all of which were identified through GC-MS analysis. Crucially, the Pluronic-modulated liposomal delivery system likely enhanced the bioavailability and targeted delivery of these hydrophobic bioactives, which work together to give purslane its hepatoprotective, anti-inflammatory, and antiapoptotic activities.

## Limitations and future directions

Our study was limited by the absence of crucial control groups, specifically one treated with PSE and another with empty PMLs, which would have helped quantify the nanocarrier’s role in enhancing bioavailability. This limitation makes it difficult to attribute the observed hepatoprotection solely to the nano-delivery system. Additionally, we lacked comprehensive pharmacokinetic data, affecting our understanding of the *in vivo* behavior of PSE-PMLs. Our model did not differentiate between direct effects on hepatocytes and indirect effects via Kupffer cells or other immune cells. Conducted in male rats to reduce hormonal variability, the study’s findings may not fully apply to females due to known sex differences in drug metabolism and liver pathophysiology. Ultimately, the study confirmed the efficacy of the whole extract only.

Future investigation is required to study the efficacy of PSE-PMLs in therapeutic post-injury models. Essential pharmacokinetic and pharmacodynamic studies should compare free PSE and PSE-PMLs to validate the nano-formulation's superiority. Additionally, studies using cell-specific knockouts or *in vitro* systems are necessary to identify direct cellular targets. The role of specific JAK2/STAT3 inhibitors combined with PSE-PMLs must be evaluated to confirm the pathway’s importance in providing protection. Investigations need to involve both male and female animals to explore potential sex-specific effects. Lastly, further mechanistic studies are required to ascertain the contributions of other PSE components to liver injury mitigation.

## Supplementary Material

Supplementary Figures S1-S2 and Table S1

## Data Availability

The datasets used and analyzed in the present study are available from the corresponding author upon reasonable request.

## Abbreviations

ALF: acute liver failure
ALT: alanine aminotransferase
ANOVA: analysis of variance
AST: aspartate aminotransferase
BAX: BCL2-associated protein x
BCL2: B-cell lymphoma 2
Caspase 3: cysteine-aspartic acid protease 3
EE%: percentage entrapment efficiency
ELISA: enzyme-linked immunosorbent assay
GC-MS: gas chromatography–mass spectrometry
H&E: hematoxylin-eosin
IFN-γ: interferon gamma
IL-4: interleukin 4
kV: kilovolt
MDA: malondialdehyde
NC: normal control
NDCs: nanodrug carriers
OD: optical density
P-F127: pluronic F127
p-JAK2: phosphorylated Janus kinase 2
p-STAT3: phosphorylated signal transducer and activator of transcription 3
p53: tumor protein p53
PDI: polydispersity index
PGC-1α: peroxisome proliferator-activated receptor-gamma coactivator 1-alpha
PPARγ: peroxisome proliferator-activated receptor-gamma
PSE-PMLs: purslane seed oil extract-Pluronic-modulated liposomes
ROS: reactive oxygen species
rpm: revolutions per minute
SD: standard deviation
SEM: standard error of the mean
SIL: silymarin
SOD: superoxide dismutase
TAA: thioacetamide
TEM: transmission electron microscopy
TMS: trimethylsilyl
VS: vesicular size
ZP: zeta potential
