# Supplementary material for: α-Linolenic acid-driven nano-liposomes from purslane seed oil modulate p-JAK2/p-STAT3 to combat acute liver failure
Source: Biosci Rep. 2026 May 8;46(5):BSR20250383. doi: 10.1042/BSR20250383 (PMC13161346; doi:10.1042/BSR20250383)
Supplement: Supplementary Figures S1-S2 and Table S1 [file BSR-2025-0383_supp.pdf]

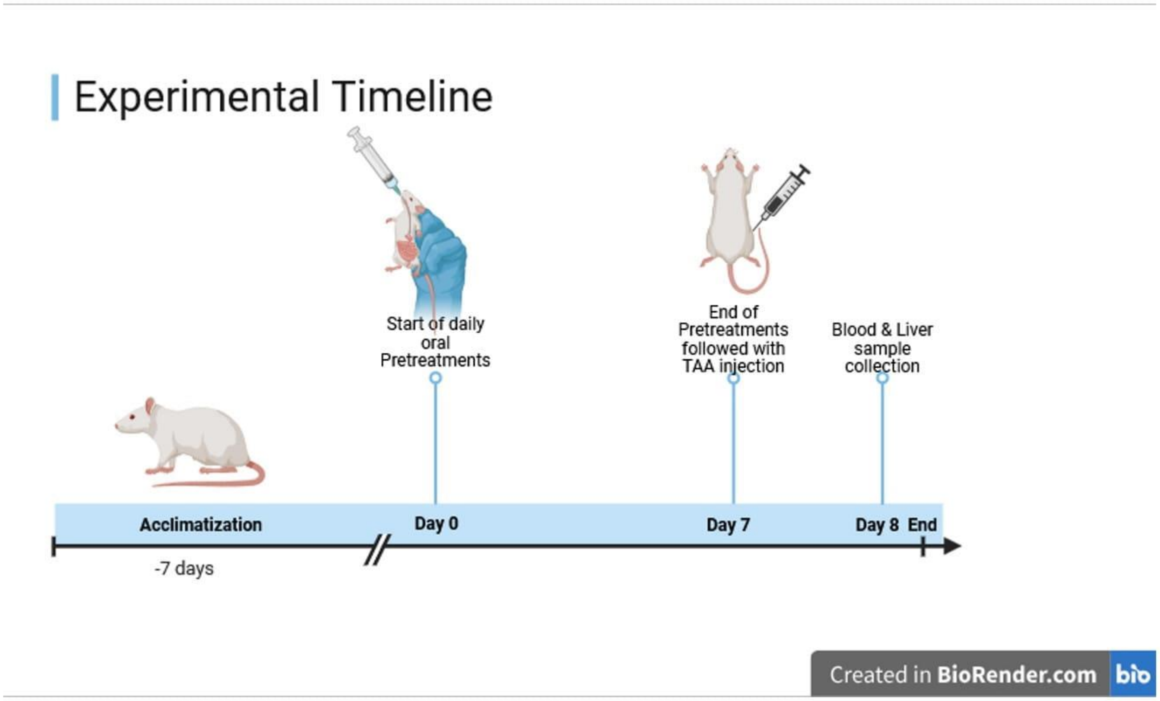

Supplementary Fig. 1 Experimental timeline. *Day -7 to Day 0: acclimatization period; Day 0 to Day 7: daily oral pretreatments; Day 7: TAA injection; Day 8 (24h post-TAA): Sample collection. TAA, thioacetamide*

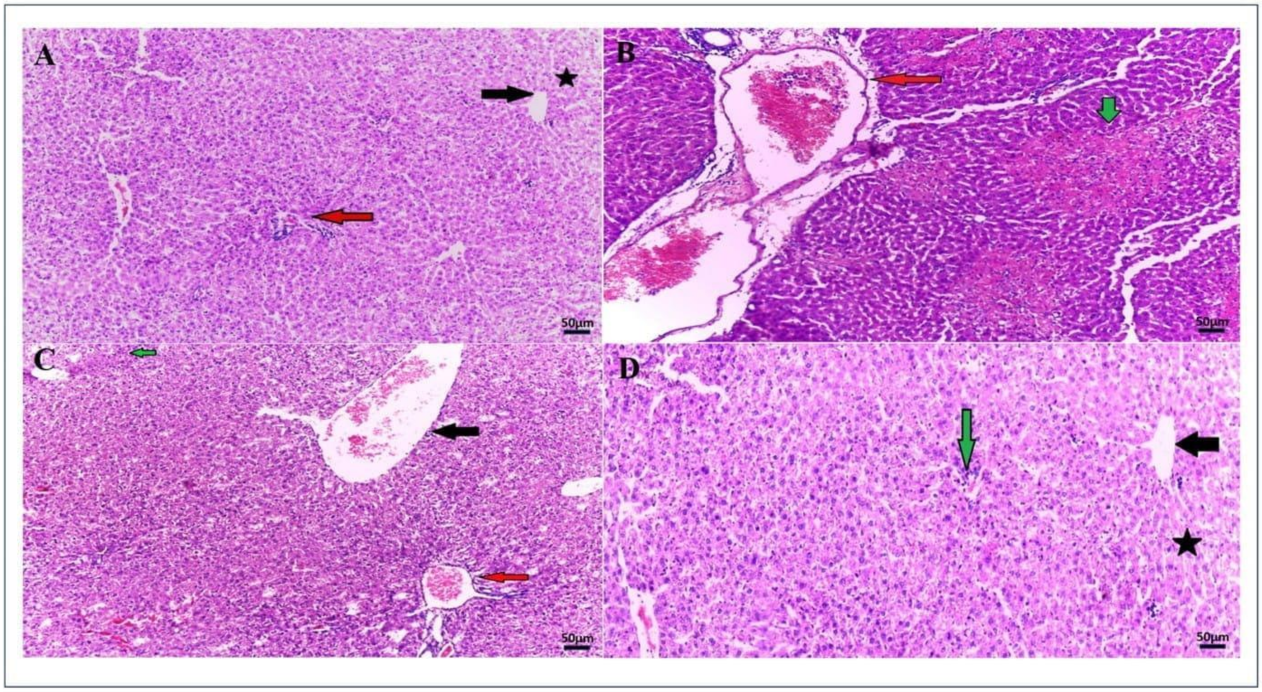

Supplementary Fig. 2 Effect of PSE-PMLs on TAA-induced histopathological alterations in liver tissues. *Representative photomicrographs of H&E-stained livers (100×) showing normal central vein (black arrow), normal portal tract (red arrow) and normal hepatocytes (star) in the normal control (NC) group (A); dilated congested portal vein (red arrow) and multiple necro-inflammatory foci (green arrow) in the TAA group (B); congested central vein (black arrow), congested portal vein (red arrow) and multiple inflammatory cells (green arrow) in the silymarin (SIL) group (C); normal central vein (black arrow), normal hepatocytes (star) and few inflammatory cells (green arrow) in the purslane seed extract-pluronic-modulated liposomes (PSE-PMLs) group (D).*

Supplementary Table 1 Correlation matrix

		T.Bil	D.Bil	AST	ALT	IL-4	IFN-γ	SOD	MDA	PGC-1α	p-JAK2	p-STAT3	PPARγ	P53	BCL2	Caspase -3
**T.Bil**	*r*															
	p															
**D.Bil**	*r*	0.96556														
	p	0.00000														
**AST**	*r*	0.98283	0.97254													
	p	0.00000	0.00000													
**ALT**	*r*	0.97796	0.97154	0.97875												
	p	0.00000	0.00000	0.00000												
**IL-4**	*r*	0.99214	0.96132	0.99023	0.97725											
	p	0.00000	0.00000	0.00000	0.00000											
**IFN-γ**	*r*	0.97501	0.97258	0.99239	0.96686	0.97370										
	p	0.00000	0.00000	0.00000	0.00000	0.00000										
**SOD**	*r*	-0.94319	-0.95154	-0.92629	-0.92741	-0.93531	-0.93527									
	p	0.00000	0.00000	0.00000	0.00000	0.00000	0.00000									
**MDA**	*r*	0.98232	0.98040	0.98948	0.98760	0.98406	0.98122	-0.93848								
	p	0.00000	0.00000	0.00000	0.00000	0.00000	0.00000	0.00000								
**PGC-1α**	*r*	-0.90588	-0.91646	-0.87887	-0.88510	-0.88720	-0.89055	0.97052	-0.90934							
	p	0.00000	0.00000	0.00000	0.00000	0.00000	0.00000	0.00000	0.00000							
**p-JAK2**	*r*	0.98191	0.98310	0.99095	0.98116	0.98122	0.98709	-0.94088	0.99694	-0.91453						
	p	0.00000	0.00000	0.00000	0.00000	0.00000	0.00000	0.00000	0.00000	0.00000						

**p-STAT3**	*r*	0.98085	0.98377	0.98947	0.97999	0.97673	0.98936	-0.94490	0.99512	-0.91848	0.99835					
	p	0.00000	0.00000	0.00000	0.00000	0.00000	0.00000	0.00000	0.00000	0.00000	0.00000					
**PPARγ**	*r*	-0.85473	-0.88183	-0.83179	-0.81602	-0.82611	-0.85819	0.91979	-0.84404	0.96374	-0.86366	-0.86935				
	p	0.00000	0.00000	0.00000	0.00000	0.00000	0.00000	0.00000	0.00000	0.00000	0.00000	0.00000				
**P53**	*r*	0.96204	0.98207	0.97837	0.96054	0.95762	0.98525	-0.93164	0.98506	-0.91585	0.99160	0.99240	-0.88497			
	p	0.00000	0.00000	0.00000	0.00000	0.00000	0.00000	0.00000	0.00000	0.00000	0.00000	0.00000	0.00000			
**BCL2**	*r*	-0.87610	-0.91659	-0.88243	-0.86672	-0.85893	-0.91290	0.94203	-0.89646	0.96510	-0.91148	-0.91868	0.96969	-0.93536		
	p	0.00000	0.00000	0.00000	0.00000	0.00000	0.00000	0.00000	0.00000	0.00000	0.00000	0.00000	0.00000	0.00000		
**Caspase-3**	*r*	0.96815	0.96894	0.97889	0.95880	0.95506	0.99140	-0.92130	0.97525	-0.90100	0.98392	0.98857	-0.88269	0.98949	-0.92736	
	p	0.00000	0.00000	0.00000	0.00000	0.00000	0.00000	0.00000	0.00000	0.00000	0.00000	0.00000	0.00000	0.00000	0.00000	

r, Pearson's correlation coefficient, significance was considered at $p < 0.05$.
